# Anti-inflammatory coumarins from *Paramignya trimera*

**DOI:** 10.1080/13880209.2017.1296001

**Published:** 2017-02-28

**Authors:** Hoang Le Tuan Anh, Dong-Cheol Kim, Wonmin Ko, Tran Minh Ha, Nguyen Xuan Nhiem, Pham Hai Yen, Bui Huu Tai, Luu Hong Truong, Vu Ngoc Long, Tran Gioi, Tran Hong Quang, Chau Van Minh, Hyuncheol Oh, Youn-Chul Kim, Phan Van Kiem

**Affiliations:** a Institute of Marine Biochemistry, Vietnam Academy of Science and Technology (VAST), Cau Giay, Hanoi, Vietnam;; b College of Pharmacy, Wonkwang University, Iksan, Republic of Korea;; c Southern Institute of Ecology, Vietnam Academy of Science and Technology (VAST), Ho Chi Minh City, Vietnam;; d Khanh Hoa Association for Conservation of Nature and Environment, Khanh Hoa, Vietnam

**Keywords:** Rutaceae, BV2 microglia, ostruthin, ninhvanin, 8-geranyl-7-hydroxycoumarin, 6-(6′,7′-dihydroxy-3′,7′-dimethylocta-2′-enyl)-7-hydroxycoumarin, 6-(7-hydroperoxy-3,7-dimethylocta-2,5-dienyl)-7-hydroxycoumarin, 6-(2-hydroxyethyl)-2,2-dimethyl-2H-1-benzopyran, luvangetin

## Abstract

**Context:***Paramignya trimera* (Oliv.) Burkill (Rutaceae) has been used to treat liver diseases and cancer. However, the anti-inflammatory effects of this medicinal plant and its components have not been elucidated.

**Objective:** This study investigated chemical constituents of the *P. trimera* stems and evaluated anti-inflammatory effects of isolated compounds.

**Materials and methods:** Cytotoxicity of isolated compounds (5–40 μM) toward BV2 cells was tested using 3-[4,5-dimethylthiazol-2-yl]-2,5-diphenyltetrazolium bromide (MTT) for 24 h. Inhibitory effects of isolated compounds (5-40 μM) on nitrite and PGE_2_ concentrations were determined using Griess reaction and PGE_2_ ELISA kit, respectively (pretreated with the compounds for 3 h and then stimulated for 18 h with LPS). Inhibitory effects of compounds (5-40 μM) on iNOS and COX-2 protein expression were evaluated by Western blot analysis (pretreated with the compounds for 3 h and then stimulated for 24 h with LPS).

**Results:** Seven coumarins were isolated and identified as: ostruthin (**1**), ninhvanin (**2**), 8-geranyl-7-hydroxycoumarin (**3**), 6-(6′,7′-dihydroxy-3′,7′-dimethylocta-2′-enyl)-7-hydroxycoumarin (**4**), 6-(7-hydroperoxy-3,7-dimethylocta-2,5-dienyl)-7-hydroxycoumarin (**5**), 6-(2-hydroxyethyl)-2,2-dimethyl-2*H*-1-benzopyran (**6**), and luvangetin (**7**). Compounds **1**–**4** and **7** inhibited NO and PGE_2_ production in LPS-stimulated BV2 cells, with IC_50_ values ranging from 9.8 to 46.8 and from 9.4 to 52.8 μM, respectively. Ostruthin (**1**) and ninhvanin (**2**) were shown to suppress LPS-induced iNOS and COX-2 protein expression.

**Discussion and conclusion:** The present study provides a scientific rationale for the use of *P. trimera* in the prevention and treatment of neuroinflammatory diseases. Ostruthin and ninhvanin might have potential therapeutic effects and should be considered for further development as new anti-neuroinflammatory agents.

## Introduction

*Paramignya trimera* (Oliv.) Burkill (Rutaceae) is a woody shrub, mostly distributed in the Southern regions of Vietnam. In the Vietnamese traditional medicine, *P. trimera* is well known as a medicinal plant used to treat liver diseases and cancer (Cuong et al. 2015; Nguyen et al. 2015). Recent pharmacological studies have shown that *P. trimera* possesses antioxidant activity and its crude methanol extract and hexane fraction display moderate cytotoxic effects toward several cancer cell lines (Nguyen et al. 2013). Previous chemical investigation of the stems and roots of *P. trimera* demonstrated the presence of several coumarins (Cuong et al. 2013, 2015). In this study, we report the isolation and structural elucidation of seven coumarins (**1**–**7**) from the methanol extract of the stems of *P. trimera*.

Microglia are the resident immune cells in the central nervous system (CNS) that play an important role in response to neuronal damage and removal of the damaged cells. In response to extracellular stimuli, including LPS, microglia are activated and produce pro-inflammatory mediators, such as nitric oxide (NO) and prostaglandin E_2_ (PGE_2_) and cytokines (Napoli & Neumann 2009). However, prolonged and excessive production of pro-inflammatory mediators by microglia appear to contribute to neuronal cell death (Gonzalez-Scarano & Baltuch 1999). Uncontrolled or aberrant activation of microglia was shown to be the cause of severe neuronal disorders, including Alzheimer’s disease, Pakinson’s disease, and Huntington’s disease (Zindler & Zipp 2010). Therefore, downregulation of pro-inflammatory mediators in microglia could be considered as an important target for the therapeutic approach of neurodegenerative diseases.

NO and PGE_2_, the inflammatory products of inducible nitric oxide synthase (iNOS) and cyclooxygenase-2 (COX-2), are considered as the major factors in the inflammatory response. Excessive production of these pro-inflammatory mediators was shown to be the cause of the neurodegenerative diseases (Nathan & Hibbs 1991; Shi et al. 2012). In order to discover anti-neurodegenerative compounds, we are looking for natural products-derived biomaterial with inhibitory effects of NO and PGE_2_ production in LPS-stimulated BV2 microglia cells. In this paper, we report the inhibitory effects of compounds **1**–**4**, **6**, and **7** on NO and PGE_2_ production and iNOS and COX-2 protein expression in LPS-stimulated BV2 microglia cells. Furthermore, the protective effects of these compounds on glutamate-induced mouse hippocampal HT22 cell injury were also reported.

## Materials and methods

### General experimental procedures

1D and 2D NMR experiments (^1^H, ^13^C, HSQC, and HMBC) were recorded on a Bruker AM500 FTNMR spectrometer: 500 MHz (^1^H NMR), 125 MHz (^13^C NMR). The electrospray ionization (ESI) mass spectra were recorded on an AGILENT 1200 LC-MSD trap spectrometer. Column chromatography (CC) was performed using silica gel (0.040–0.063 mm, Merck) and RP-18 resins (30–50 μm, Fuji Silysia Chemical Ltd.). Thin layer chromatography (TLC) was performed on DC-Alufolien 60 F_254_ (1.05715, Merck) and RP18 F_254s_ (Merck) plates.

### Plant material

The stems of *P. trimera* were collected in Khanh Hoa province, Vietnam during December 2013, and identified by one of the authors, Dr. Luu Hong Truong. A voucher specimen (NCCT-TR.01) was deposited at the Herbarium of the Institute of Marine Biochemistry, VAST.

### Extraction and isolation

The dried stems of *Paramignya trimera* (3 kg) were ground and extracted with MeOH under sonication at room temperature. After concentration under reduced pressure, the MeOH extract (150 g) was suspended in water and then partitioned successively with CHCl_3_ and EtOAc to give CHCl_3_-, EtOAc-, and water-soluble fractions. The CHCl_3_-soluble fraction (60 g) was subjected to fractionation over silica gel, eluting with EtOAc in *n*-hexane (1-100%, step-wise) to give fractions TR1A-E. Fraction TR1A was separated by column chromatography (CC) over silica gel, using CH_2_Cl_2_-MeOH (26:1, v/v) as eluent to yield **5** (8 mg). Fraction TR1C was subjected to silica gel CC and eluted with *n*-hexane-EtOAc (7:1, v/v) to provide **1** (50 mg) and **2** (20 mg). Fraction TR1E was chromatographed over a silica gel column, eluting with *n*-hexane-acetone (9:2, v/v), and further purified by silica gel CC, using *n*-hexane-acetone (3:1, v/v) as eluent to give **6** (8 mg) and **7** (8 mg). The EtOAc-soluble fraction (40 g) was subjected to reversed phase (RP) C_18_ CC, eluting with a gradient of acetone in water (1:3–2:1, v/v) to give fractions TR2A-C. Fraction TR2A was separated by silica gel CC, using CH_2_Cl_2_-MeOH (15:1, v/v) as eluent to provide **4** (8 mg). Fraction TR2B was separated by silica gel CC, eluting with *n*-hexane-acetone (3:1, v/v) to give **3** (10 mg).

#### Ostruthin (**1**)

White, amorphous powder; ^1^H NMR (CDCl_3_, 500 MHz): δ 6.23 (d, *J* = 9.5 Hz, H-3), 7.65 (d, *J* = 9.5 Hz, H-4), 7.20 (s, H-5), 7.07 (s, H-8), 3.39 (d, *J* = 7.0 Hz, H_2_-1′), 5.34 (t, *J* = 7.0 Hz, H-2′), 2.10 (m, H_2_-4′), 2.12 (m, H_2_-5′), 5.10 (t, *J* = 6.5 Hz, H-6′), 1.68 (s, H_3_-8′), 1.60 (s, H_3_-9′), 1.73 (s, H_3_-10′); ^13^C NMR (CDCl_3_, 125 MHz): δ 162.6 (C-2), 111.9 (C-3), 144.5 (C-4), 128.1 (C-5), 126.1 (C-6), 158.9 (C-7), 103.0 (C-8), 154.1 (C-9), 112.1 (C-10), 28.1 (C-1′), 120.9 (C-2′), 138.3 (C-3′), 39.7 (C-4′), 26.5 (C-5′), 123.9 (C-6′), 131.7 (C-7′), 25.7 (C-8′), 17.7 (C-9′), 16.1 (C-10′).

#### Ninhvanin (**2**)

White, amorphous powder; ^1^H NMR (CDCl_3_, 500 MHz): δ 6.22 (d, *J* = 9.5 Hz, H-3), 7.60 (d, *J* = 9.5 Hz, H-4), 6.95 (s, H-5), 3.37 (d, *J* = 7.5 Hz, H_2_-1′), 5.32 (t, *J* = 7.5 Hz, H-2′), 2.09 (m, H_2_-4′), 2.13 (m, H_2_-5′), 5.11 (t, *J* = 6.5 Hz, H-6′), 1.68 (s, H_3_-8′), 1.60 (s, H_3_-9′), 1.71 (s, H_3_-10′), 4.10 (s, 8-OCH_3_); ^13^C NMR (CDCl_3_, 125 MHz): δ 160.7 (C-2), 112.2 (C-3), 144.4 (C-4), 122.3 (C-5), 125.2 (C-6), 150.2 (C-7), 133.1 (C-8), 145.3 (C-9), 112.2 (C-10), 27.5 (C-1′), 120.8 (C-2′), 137.4 (C-3′), 39.6 (C-4′), 26.5 (C-5′), 124.0 (C-6′), 131.4 (C-7′), 25.6 (C-8′), 17.6 (C-9′), 16.0 (C-10′), 61.6 (8-OCH_3_).

#### 8-Geranyl-7-hydroxycoumarin (**3**)

White, amorphous powder; ^1^H NMR (CD_3_OD, 500 MHz): δ 6.18 (d, *J* = 9.5 Hz, H-3), 7.85 (d, *J* = 9.5 Hz, H-4), 7.31 (d, *J* = 8.5 Hz, H-5), 6.83 (d, *J* = 8.5 Hz, H-6), 3.53 (d, *J* = 7.0 Hz, H_2_-1′), 5.26 (t, *J* = 7.0 Hz, H-2′), 1.98 (m, H_2_-4′), 2.06 (m, H_2_-5′), 5.03 (t, *J* = 7.0 Hz, H-6′), 1.57 (s, H_3_-8′), 1.53 (s, H_3_-9′) 1.85 (s, H_3_-10′); ^13^C NMR (CD_3_OD, 125 MHz): δ 164.0 (C-2), 111.4 (C-3), 146.6 (C-4), 127.6 (C-5), 113.8 (C-6), 161.1 (C-7), 117.0 (C-8), 154.8 (C-9), 113.1 (C-10), 22.6 (C-1′), 122.8 (C-2′), 136.5 (C-3′), 40.8 (C-4′), 27.5 (C-5′), 125.3 (C-6′), 132.0 (C-7′), 25.7 (C-8′), 17.6 (C-9′), 16.4 (C-10′).

#### 6-(6′,7′-Dihydroxy-3′,7′-dimethylocta-2′-enyl)-7-hydroxycoumarin (**4**)

White, amorphous powder; HRESIMS: *m/z* 331.1571 [M-H]^-^ (calcd. for C_19_H_23_O_5_, 331.1545); ^1^H NMR (CD_3_OD, 500 MHz): δ 6.17 (d, *J* = 9.5 Hz, H-3), 7.83 (d, *J* = 9.5 Hz, H-4), 7.30 (s, H-5), 6.72 (s, H-8), 3.34 (d, *J* = 7.5 Hz, H_2_-1′), 5.42 (t, *J* = 7.5 Hz, H-2′), 2.12 (m, H-4′a), 2.34 (m, H-4′b), 1.43 (m, H-5′a), 1.81 (m, H-5′b), 3.29 (dd, *J* = 1.5, 10.5 Hz, H-6′), 1.18 (s, H_3_-8′), 1.15 (s, H_3_-9′), 1.75 (s, H_3_-10′); ^13^C NMR (CD_3_OD, 125 MHz): δ 164.0 (C-2), 112.0 (C-3), 146.3 (C-4), 129.4 (C-5), 127.9 (C-6), 160.8 (C-7), 102.6 (C-8), 155.4 (C-9), 112.9 (C-10), 28.6 (C-1′), 123.1 (C-2′), 137.8 (C-3′), 37.8 (C-4′), 30.6 (C-5′), 78.9 (C-6′), 73.7 (C-7′), 25.7 (C-8′), 24.9 (C-9′), 16.2 (C-10′).

#### 6-(7-Hydroperoxy-3,7-dimethylocta-2,5-dienyl)-7-hydroxycoumarin (**5**)

White, amorphous powder; ^1^H NMR (CD_3_OD, 500 MHz): δ 6.14 (d, *J* = 9.5 Hz, H-3), 7.83 (d, *J* = 9.5 Hz, H-4), 7.29 (s, H-5), 6.71 (s, H-8), 3.37 (d, *J* = 7.5 Hz, H_2_-1′), 5.42 (t, *J* = 7.5 Hz, H-2′), 2.80 (d, *J* = 3.5 Hz, H_2_-4′), 5.66 (overlapped, H-5′ and H-6′), 1.31 (s, H_3_-8′ and H_3_-9′), 1.73 (s, H_3_-10′); ^13^C NMR (CD_3_OD, 125 MHz): δ 164.2 (C-2), 111.5 (C-3), 146.3 (C-4), 129.3 (C-5), 128.0 (C-6), 162.0 (C-7), 102.7 (C-8), 155.6 (C-9), 112.6 (C-10), 28.6 (C-1′), 123.9 (C-2′), 136.6 (C-3′), 43.6 (C-4′), 129.4 (C-5′), 137.0 (C-6′), 82.4 (C-7′), 24.9 (C-8′ and C-9′), 16.2 (C-10′).

#### 6-(2-Hydroxyethyl)-2,2-dimethyl-2*H*-1-benzopyran (**6**)

White, amorphous powder; ^1^H NMR (CD_3_OD, 500 MHz): δ 5.60 (d, *J* = 10.0 Hz, H-3), 6.28 (d, *J* = 10.0 Hz, H-4), 6.82 (d, *J* = 2.5 Hz, H-5), 6.94 (dd, *J* = 2.5, 8.5 Hz, H-7), 6.71 (d, *J* = 8.5 Hz, H-8), 2.75 (t, *J* = 6.5 Hz, H_2_-11), 3.79 (t, *J* = 6.5 Hz, H_2_-12), 1.42 (s, 2-(CH_3_)_2_); ^13^C NMR (CD_3_OD, 125 MHz): δ 76.0 (C-2), 130.9 (C-3), 122.1 (C-4), 126.7 (C-5), 130.4 (C-6), 129.4 (C-7), 116.3 (C-8), 151.5 (C-9), 121.2 (C-10), 38.3 (C-11), 63.7 (C-12), 27.9 (2-(CH_3_)_2_).

#### Luvangetin (**7**)

White, amorphous powder; ^1^H NMR (CDCl_3_, 500 MHz): δ 6.24 (d, *J* = 9.5 Hz, H-3), 7.57 (d, *J* = 9.5 Hz, H-4), 6.82 (s, H-5), 6.33 (d, *J* = 10.0 Hz, H-1′), 5.71 (d, *J* = 10.0 Hz, H-2′), 1.51 (s, H_3_-4′ and H_3_-5′), 3.98 (s, 8-OCH_3_); ^13^C NMR (CDCl_3_, 125 MHz): δ 160.6 (C-2), 113.0 (C-3), 143.5 (C-4), 113.2 (C-4a), 119.0 (C-5 and C-6), 149.3 (C-7), 135.6 (C-8), 148.3 (C-8a), 121.0 (C-1′), 131.2 (C-2′), 77.7 (C-3′), 28.2 (C-4′ and C-5′), 61.4 (8-OCH_3_).

### Cell culture and viability assay

BV2 microglia cells were obtained from Prof. Hyun Park at Wonkwang University (Iksan, Korea) and mouse hippocampal HT22 cells were received from Dr. Inhee-Mook Seoul National University (Seoul, Korea). The cells were maintained at 5 × 10^5^ cells/mL in 100-mm dishes in DMEM supplemented with 10% heat-inactivated FBS, penicillin G (100 units/mL), streptomycin (100 μg/mL), and l-glutamine (2 mM), and were incubated at 37 °C in a humidified atmosphere containing 5% CO_2_ and 95% air. The effects of the various experimental modulations on cell viability were evaluated by determining the mitochondrial reductase function based on reduction of the tetrazolium salt, 3-[4,5-dimethylthiazol-2-yl]-2,5-diphenyltetrazolium bromide (MTT) to a formazan crystal (Berridge & Tan 1993). The formation of formazan is proportional to the number of functional mitochondria in the living cells. For the determination of cell viability, cells (2 × 10^4^ cells/200 μL in each well of the 96-well plates) were incubated with MTT at a final concentration of 0.5 mg/mL for 4 h. The formazan formed was dissolved in acidic 2-propanol, and the optical density was measured at 590 nm. The optical density of the formazan formed in the control (untreated) cells was considered as 100% viability. For determination of cytoprotective effects, HT22 cells (5 × 10^5^ cells/mL in DMEM medium) were treated with compounds in the presence of 20 mM glutamate and incubated for indicated times and cell viability by MTT assay.

### Nitrite (NO production) determination

The production of nitrite, the stable end product of NO oxidation, was used as a measure of iNOS activity. The nitrite present in the conditioned media was determined by a method based on the Griess reaction (Titheradge 1998). Briefly, the cells were pretreated for 3 h with different concentrations of compounds and then stimulated for 18 h with LPS (1 μg/mL). Each cell supernatant (100 μL) was mixed with an equal volume of the Griess reagent (Solution A: 222488; Solution B: S438081, Sigma), and the absorbance of the mixture at 525 nm was measured using an ELISA plate reader. The natural product-derived anti-inflammatory agent, butein was used as the positive control.

### PGE_2_ assay

BV2 microglial cells were cultured in 24-well plates, pre-incubated for 3 h with different concentrations of compounds, and then stimulated for 18 h with LPS (Sigma-Aldrich). Supernatant of the culture media (100 μL) was collected to determine the PGE_2_ concentration using an ELISA kit (R & D Systems). Butein was used as the positive control.

### Western blot analysis

BV2 cells were lysed in 20 mM Tris-HCl buffer (pH 7.4) containing a protease inhibitor mixture (0.1 mM PMSF, 5 mg/mL aprotinin, 5 mg/mL pepstatin A, and 1 mg/mL chymostatin). The protein concentration was determined using the Lowry protein assay kit (P5626; Sigma). An equal amount of protein from each sample was resolved using 7.5% sodium dodecyl sulfate-polyacrylamide gel electrophoresis (SDS-PAGE) and then electrophoretically transferred to the Hybond enhanced chemiluminescence (ECL) nitrocellulose membrane (Bio-Rad Laboratories, Hercules, CA, USA). The membrane was blocked using 5% skim milk and subsequently incubated with the primary antibody (Santa Cruz Biotechnology, Inc., Santa Cruz, CA) and horseradish peroxidase-conjugated secondary antibody, followed by ECL detection (Amersham Corp., Arlington Heights, IL).

### Statistical analysis

Data expressed as the mean ± standard deviation (SD) of at least three independent experiments. Three or more groups were compared using one-way analysis of variance followed by the Newman-Keuls *post hoc* test. Statistical analysis was performed using GraphPad Prism software, version 3.03 (GraphPad Software Inc, San Diego, CA).

## Results and discussion

**Figure 1.**
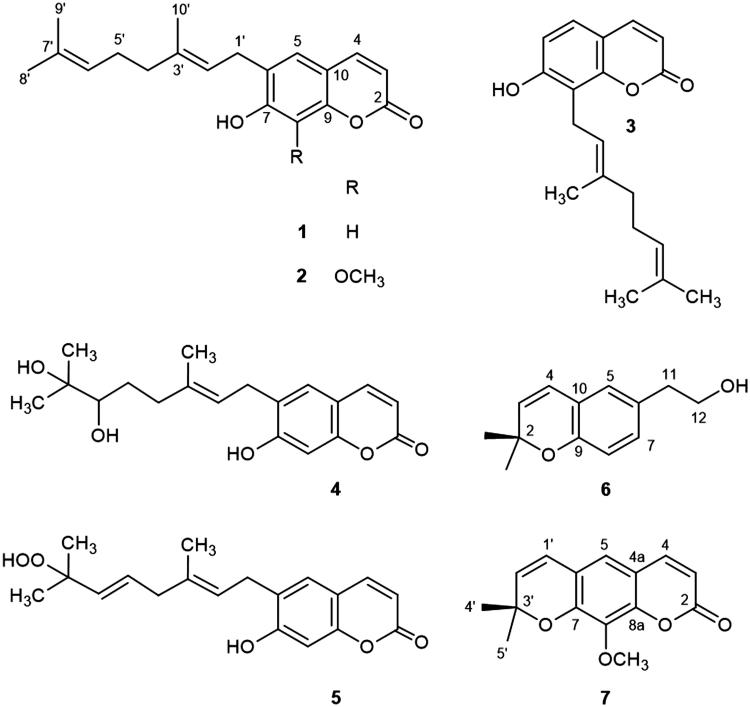
Chemical structures of compounds **1**–**7** from *P. trimera*.

A MeOH extract of *P. trimera* was suspended in H_2_O and successively partitioned with CHCl_3_ and EtOAc to give CHCl_3_-, EtOAc-, and H_2_O-soluble fractions. The CHCl_3_- and EtOAc-soluble fractions were subjected to multiple chromatographic steps over silica gel and reversed phase C_18_, yielding compounds **1**–**7.** The structures of these compounds were identified as: ostruthin (**1**) (Liu et al. 2005), ninhvanin (**2**) (Cuong et al. 2013), 8-geranyl-7-hydroxycoumarin (**3**) (Rashid et al. 1992), 6-(6′,7′-dihydroxy-3′,7′-dimethylocta-2′-enyl)-7-hydroxycoumarin (**4**) (Nguyen et al. 2016), 6-(7-hydroperoxy-3,7-dimethylocta-2,5-dienyl)-7-hydroxycoumarin (**5**) (Rashid et al. 1992), 6-(2-hydroxyethyl)-2,2-dimethyl-2H-1-benzopyran (**6**) (Wattanapiromsakul & Waterman 2000), and luvangetin (**7**) (Patra & Mitra 1981) by analysis of the 1D and 2D NMR spectroscopic data and comparison with the known values (Figure 1). It is noted that this is the first time to report the isolation of compounds **3**–**5** and **7** from the *Paramignya* genus and the NMR data for compound **4**. Coumarin is a secondary phytochemical that belongs to the benzopyrone chemical class. Coumarin has been shown to possess various pharmacological properties such as anti-inflammatory, anticoagulant, antibacterial, antifungal, antiviral, anticancer, antihypertensive, antitubercular, anticonvulsant, antiadipogenic, antihyperglycemic, antioxidant, and neuroprotective activities (Venugopala et al. 2013). With the broad range of pharmacological effects, along with low toxicity and occurrence in many herbal remedies, coumarins appear as the promising target for discovering and developing novel therapeutic agents. Previous studies on the pharmacological effects of the isolated compounds have shown that ostruthin (**1**) is cytotoxic toward two human pancreatic cancer cell lines, including PANC-1 and PSN-1 cells in nutrient-deprived medium (Li et al. 2012), this compound was shown to inhibit vascular smooth muscle cell proliferation (Joa et al. 2011), inhibit acetylcholinesterase activity (Urbain et al. 2005), and display antimycobacterial activity (Schinkovitz et al. 2003); luvangetin (7) was shown to have significant protection against pylorus-ligated and aspirin-induced gastric ulcers in rats and cold restraint stress-induced gastric ulcers in rats and guinea pigs (Goel et al. 1997). However, there are no reports on the anti-inflammatory effects of the compounds **1**–**7** so far. Therefore, the anti-inflammatory effects of the isolated compounds were evaluated through inhibition of NO and PGE_2_ production and attenuation of iNOS and COX-2 protein expression in LPS-stimulated BV2 cells. Because compound **5** was decomposed, so it was excluded out of the evaluation of biological effects of the isolated compounds.

Microglia are important immune cells in the CNS because microglial activation initiate an inflammatory cascade and is known to trigger various neurodegenerative diseases. In this context, the control of microglial activation has been postulated as a putative target in the treatment of neurodegenerative diseases (Dheen et al. 2007). Therefore, we use LPS-stimulated BV2 microglia as a screening system to look for anti-neuroinflammatory natural compounds.

To exclude the possibility that our results could be misinterpreted by cytotoxic effects, we used the MTT assay to evaluate the viability of BV2 cells treated with various concentrations (5–40 μM) of isolated compounds. As the result, all of the tested compounds did not exhibit significant cytotoxicity in BV2 microglia cells at the tested concentrations (Figure S1, Supplementary material). Thus, for all subsequent experiments, the concentrations of the tested compounds were used within the range of 5–40 μM.

**Figure 2.**
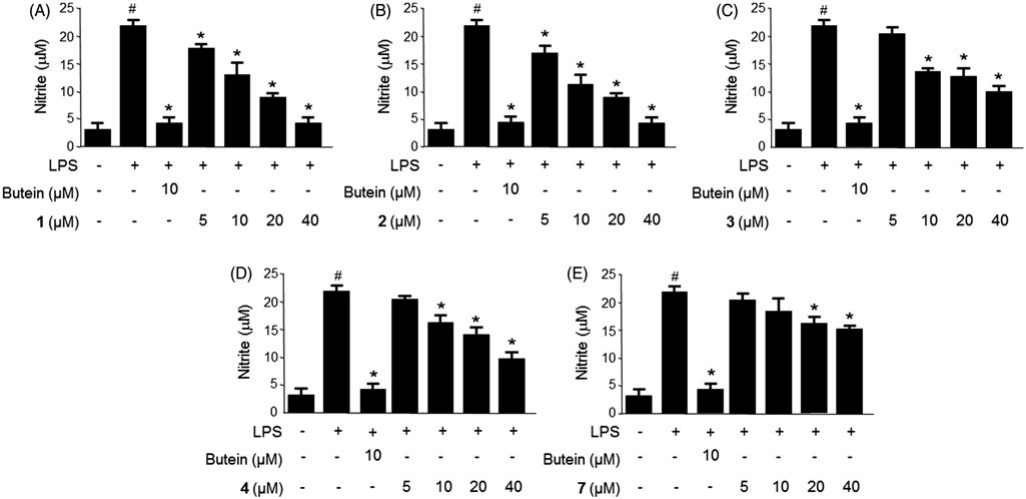
Effects of compounds **1**–**4**, and **7** on nitrite production in LPS-stimulated BV2 microglia (A−E). Cells were pretreated for 3 h with the indicated concentrations of the compounds, then stimulated for 18 h with LPS (1 μg/mL). The concentrations of nitrite were determined using a Griess reaction. Data represent the mean ± S.D. of three experiments. #*p* < 0.05, as compared with the control group; *p < 0.05, as compared with the group treated with LPS only. Butein was used as the positive control.

**Figure 3.**
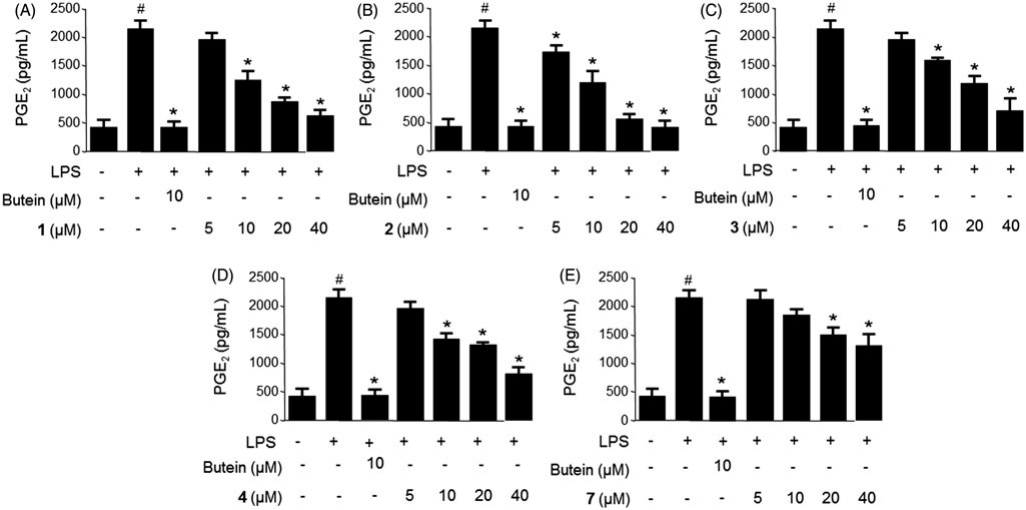
Effects of compounds **1**–**4**, and **7** on PGE_2_ production in LPS-stimulated BV2 microglia (A−E). Cells were pretreated for 3 h with the indicated concentrations of the compounds and then stimulated for 18 h with LPS (1 μg/mL). The concentrations of PGE_2_ were determined using a PGE_2_ ELISA kit. Data represent the mean ± S.D. of three experiments. #*p* < 0.05, as compared with the control group; *p < 0.05, as compared with the group treated with LPS only. Butein was used as the positive control.

The microglia stimulants, including LPS were shown to induce neurotoxicity through the generation of pro-inflammatory mediators, such as nitric oxide (NO), prostaglandin E_2_ (PGE_2_) and pro-inflammatory cytokines (e.g. tumor necrosis factor-α), interleukins, and cytotoxic factors (Hammond et al. 1999; Brown & Neher 2010; Kang et al. 2012). Therefore, the levels of NO and PGE_2_ production in the LPS-stimulated migroglia may reflect the degree of inflammation and provide information to investigate the anti-inflammatory effects of the natural compounds (Chun et al. 2012). The anti-inflammatory effects of compounds **1**–**4**, **6**, and **7** were initially evaluated through inhibition of NO and PGE_2_ production in LPS-stimulated BV2 cells. BV2 cells were pretreated for 3 h with different concentrations (5–40 μM) of compounds **1**–**4**, **6**, and **7** followed by treatment with LPS (1 μg/mL) for 18 h. As shown in Figure 2, treatment of BV2 cells with LPS triggered an approximate seven-fold increase in nitrite concentration compared with that of the untreated group. When pretreated the cells with compounds **1**–**4**, and **7** for 3 h, the production of NO, as indicated by the nitrite concentration, was decreased in a dose-dependent manner, with IC_50_ values ranging from 9.8–46.8 μM (Table 1). The PGE_2_ concentration was also increased approximately eight-fold compared with that of the untreated group when treating cells with LPS (Figure 3). However, pretreatment of the cells with compounds **1**–**4**, and **7** for 3 h decreased the production of PGE_2_ in a dose-dependent manner, with IC_50_ values in the range of 9.4–52.8 μM (Table 1). Compound **6** displayed no inhibition on NO and PGE_2_ production at the tested concentration range. Considering the inhibitory effects of compounds **1**–**4,** and **7** on the NO and PGE_2_ production, we next investigated the inhibitory effects of compounds **1** and **2** on iNOS and COX-2 protein expression in LPS-stimulated BV2 cells.

**Table 1.** Inhibitory effects of compounds **1**–**4**, **6**, and **7** on NO and PGE_2_ production.

	IC_50_ (μM)	
Compound	NO	PGE_2_
**1**	12.3 ± 0.6	13.4 ± 0.7
**2**	9.8 ± 0.5	9.4 ± 0.5
**3**	36.8 ± 1.8	34.7 ± 1.7
**4**	36.5 ± 1.8	32.1 ± 1.6
**6**	>80^a^	>80^a^
**7**	46.8 ± 2.3	52.8 ± 2.6

a A compound is considered inactive with IC_50 _>_ _80 μM.

The values are mean ± SD (*n* = 3).

**Figure 4.**
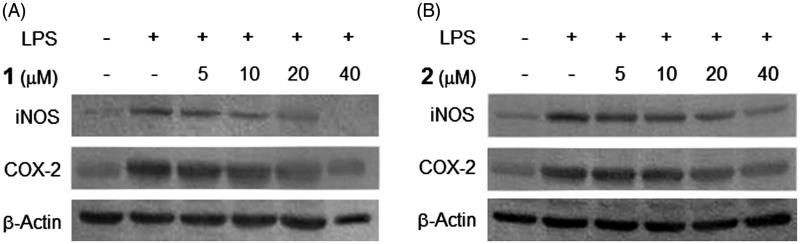
Effects of compounds **1** and **2** on iNOS and COX-2 protein expression in LPS-stimulated BV2 microglia. Cells were pretreated for 3 h with indicated concentrations of compounds **1** and **2**, then stimulated for 24 h with LPS (1 μg/mL). Western blot analyses (A and B) were performed as described in ‘Materials and methods’ section.

NO and PGE_2_ are pro-inflammatory mediators produced by their inducible enzymes iNOS and COX-2, respectively. These mediators play key roles in the activation of macrophages during the inflammation response (Korhonen et al. 2005; Zhou et al. 2008). Therefore, suppression of NO and PGE_2_ production by inhibition of iNOS and COX-2 protein expression could be considered as a therapeutic approach for anti-inflammatory diseases. The effects of compounds **1** and **2** on iNOS and COX-2 protein expression in BV2 cells were assessed by Western blot analysis. As shown in Figure 4, the expression of iNOS and COX-2 proteins was significantly up-regulated in response to LPS (1 μg/mL), however, compounds **1** and **2** suppressed the LPS-induced expression of iNOS and COX-2 in a concentration-dependent manner, respectively. These results suggest that these compounds inhibit pro-inflammatory mediators through suppressing iNOS and COX-2 protein expression in LPS-stimulated BV2 cells. The housekeeping protein, β-actin was shown to be unchanged by the presence of compounds **1** and **2** at the same concentrations.

NF-κB, a major transcription factor, plays a key role in the regulation of the inflammatory response. It is well known that NF-κB is mainly regulated by Toll like receptor 4 (TLR4) (Fitzgerald et al. 2003; Oeckinghaus et al. 2011). Down-stream of CD14, MyD88, TRIF, TRAF6, and TAK1 also conduct an important role in the regulation of phosphorylation of IκB and NF-kB heterodimers (p50 and p65) (Zhang & Ghosh 2001). Once stimulated by inflammatory signals such as LPS, IL-1β, and TNF-α, IκB is phosphorylated and degraded resulting in liberation of NF-κB (Karin & Ben-Neriah 2000; Lappas et al. 2002). Afterward, NF-κB p50/p65 heterodimers translocate to the nucleus and bind to the κB sites to control the transcription of the target genes, triggering expression of pro-inflammatory enzymes and cytokines such as iNOS, COX-2, TNF-α, IL-β, etc. Subsequently, NO and PGE_2_ are produced by iNOS and COX-2, respectively. Accordingly, when iNOS and COX-2 protein expression is inhibited by the tested compounds, production levels of NO and PGE_2_ are also decreased. In this study, we investigated the anti-inflammatory effects of the compounds via regulation of NO and PGE_2_ production. The mechanism of action of the compounds on NF-κB signalling may be conducted in a further study.

The cytoprotective effects of compounds **1**–**4**, **6**, and **7** were evaluated on the glutamate-induced toxicity in mouse hippocampal HT22 cells. However, all of the isolated compounds did not show any significant protection.

In summary, chemical investigation of the stems of *P. trimera* resulted in the isolation and identification of seven coumarin derivatives, including: ostruthin (**1**), ninhvanin (**2**), 8-geranyl-7-hydroxycoumarin (**3**), 6-(6′,7′-dihydroxy-3′,7′-dimethylocta-2′-enyl)-7-hydroxycoumarin (**4**), 6-(7-hydroperoxy-3,7-dimethylocta-2,5-dienyl)-7-hydroxycoumarin (**5**), 6-(2-hydroxyethyl)-2,2-dimethyl-2*H*-1-benzopyran (**6**), and luvangetin (**7**). Although some pharmacological effects of several coumarins have been reported as aforementioned, their anti-inflammatory effects in LPS-stimulated BV2 microglia and protective activity against glutamate-induced toxicity in HT22 cells have yet to be elucidated. In addition to the first report of compounds **3**–**5** and **7** from the *Paramignya* genus and the NMR data of compound **4**, the present study reported the anti-inflammatory effects of the isolated coumarins for the first time. The results demonstrated that compounds **1**–**4**, and **7** inhibited the NO and PGE_2_ production in LPS-stimulated BV2 cells in a dose-dependent manner. Furthermore, the inhibitory effects of compounds **1** and **2** on NO and PGE_2_ production were confirmed by the inhibition of LPS-induced iNOS and COX-2 expression. These results provide a scientific rationale for the use of *P. trimera* in the prevention and treatment of neuroinflammatory diseases and suggest that ostruthin (**1**) and ninhvanin (**2**) should be considered as lead compounds for further development as new anti-neuroinflammatory agents.

## Supplementary Material

Phan_Van_Kiem_et_al_supplemental_content.zip
