# Supplementary material for: Anti-inflammatory coumarins from Paramignya trimera
Source: Pharm Biol. 2017 Feb 28;55(1):1195–201. doi: 10.1080/13880209.2017.1296001 (PMC6130569; doi:10.1080/13880209.2017.1296001)
Supplement: Phan_Van_Kiem_et_al_supplemental_content.zip [file IPHB_A_1296001_SM8609.zip › Phan Van Kiem et al supplemental content.docx]

**Supplementary material**

**Anti-inflammatory coumarins from *Paramignya trimera***

Hoang Le Tuan Anh^a,*^, Dong-Cheol Kim^b,*^, Wonmin Ko^b^, Tran Minh Ha^a,b^, Nguyen Xuan Nhiem^a^, Pham Hai Yen^a^, Bui Huu Tai^a^, Luu Hong Truong^c^, Vu Ngoc Long^c^, Tran Gioi^d^, Tran Hong Quang^a,b^, Chau Van Minh^a^, Hyuncheol Oh^b^, Youn-Chul Kim^b^, and Phan Van Kiem^a^

^a^Institute of Marine Biochemistry, Vietnam Academy of Science and Technology (VAST), 18 Hoang Quoc Viet, Cau Giay, Hanoi, Vietnam

^b^College of Pharmacy, Wonkwang University, Iksan 570-749, Korea

^c^Southern Institute of Ecology, Vietnam Academy of Science and Technology (VAST), 01 Mac Dinh Chi, Ho Chi Minh City, Vietnam

^d^Khanh Hoa Association for Conservation of Nature and Environment, Khanh Hoa, Vietnam

**Contact:**

Phan Van Kiem

E-mail: phankiem@yahoo.com

Address: Institute of Marine Biochemistry, Vietnam Academy of Science and Technology, Hanoi, Vietnam

Youn-Chul Kim

E-mail: yckim@wku.ac.kr

Address : College of Pharmacy, Wonkwang University, Iksan 570-749, Korea

^*^Both authors contributed equally to this work and should be considered as co-first authors.


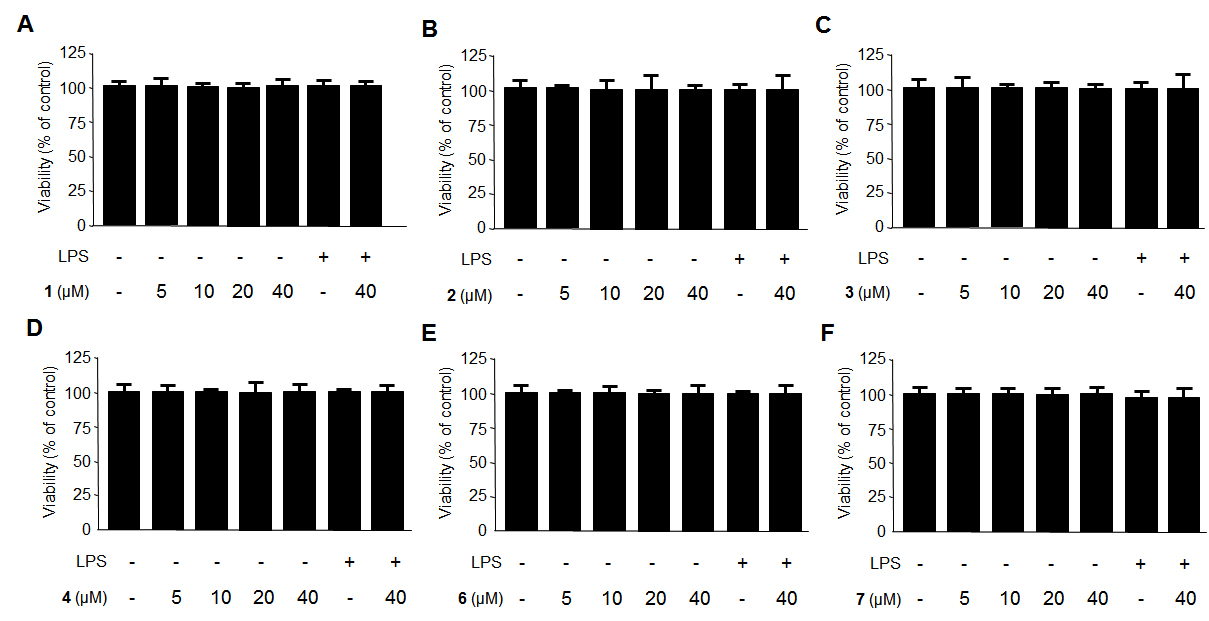


**Figure S1.** Effects of compounds **1-4, 6**, and **7** on BV2 cell viability (A-F). BV2 microglia were incubated for 24 h with various concentrations of compounds **1-4, 6**, and **7** (5-40 μM). Cell viability was determined as described in the Materials and methods. Data represent the mean values of three experiments ±SD.
